# Effects of Rock Type and Food Availability on Bioerosion by the Purple Sea Urchin, *Strongylocentrotus purpuratus*

**DOI:** 10.1093/icb/icae060

**Published:** 2024-06-03

**Authors:** Lukas U Troha, Carla A Narvaez, Michael P Russell

**Affiliations:** Department of Biology, Villanova University, Villanova, PA 19085, USA; Marine Resources Division, South Carolina Department of Natural Resources, Charleston, SC 29412, USA; Department of Biology, Villanova University, Villanova, PA 19085, USA; Department of Biology, Rhode Island College, Providence, RI 02908, USA; Department of Biology, Villanova University, Villanova, PA 19085, USA

## Abstract

Purple sea urchins (*Strongylocentrotus purpuratus*) profoundly impact nearshore rocky coasts through their feeding habits. Their intense grazing sculpts substrates through bioerosion using their teeth and spines and controls the alternative stable state dynamic between kelp bed and urchin barrens. These states have contrasting food availability for sea urchins, with abundant food in kelp beds and scarce food in barren grounds. However, the relationship between food availability and bioerosion is unknown. We predicted that when kelp is available, it would ameliorate the action of teeth on the substrate. Our 11-week long, 2 × 2 factorial experiment, crossed community state (kelp present vs absent) and rock type (sandstone vs mudstone). We also quantified the contribution of spine abrasion to bioerosion on the two rock types. The bioerosion rates did not differ between treatments with and without kelp. Although there was no significant difference in net bioerosion between the rock types, there was a large difference between the proportion of bioerosion from teeth vs spine abrasion. Approximately a third of the sandstone bioerosion was from spines whereas less than 2% of mudstone bioerosion could be attributed to spines. As anticipated, growth of sea urchins fed kelp *ad-libitum* was higher than food-limited sea urchins. Surprisingly, sea urchins on mudstone (which has a higher organic component) grew faster than sea urchins on sandstone. Although bioerosion rates may not differ on a per-urchin basis between community states, the sea urchin population densities between kelp beds and urchin barrens likely causes a difference in net bioerosion between these communities. Our results point to the importance of lithology on the mechanics of sea urchin bioerosion. Differences in texture, grain size, and hardness of rock substrates undoubtedly contribute to bioerosion rates and dynamics.

## Introduction

Sea urchins are key members of temperate rocky reef and coral communities worldwide. The purple sea urchin, *Strongylocentrotus purpuratus*, is abundant in the intertidal and shallow subtidal of the northeast Pacific from Baja California Norte to Southeast Alaska (Ricketts et al. 1985; Pearse 2006; Lester et al. 2007; Pearse et al. 2007). In the intertidal, *S. purpuratus* frequently inhabits boreholes or pits in the rocky substrate (Verling et al. 2004). These pits are a product of bioerosion, providing refuge from predators (Clemente et al. 2013), influencing behavior and morphology (Hernandez and Russell 2010), and even providing habitat for various organisms when unoccupied by sea urchins (Chanket and Wanngkulangkul 2019).

Russell et al. (2018) hypothesized that *S. purpuratus* primarily excavate these intertidal pits with their teeth, which are strong enough to bioerode plastic (Porter et al. 2019) and even steel (Lawrence and Sammarco 1982). Echinoids exhibit a five-fold symmetrical tooth structure (Ma et al. 2007), and they continually grow, and self-sharpen by scraping against the substrate (Wang et al. 1997; Killian et al. 2011). The scraping incidentally abrades the substrate and the sea urchins ingest the eroded particles, which are detectable in their feces (Russell et al. 2018). It has long been assumed that sea urchins sculpt pits with both their teeth and spines (Fewkes 1890; Otter 1932; Ricketts et al. 1985; Solovjev and Markov 2013), but distinguishing the roles of spinal and tooth abrasion on bioerosion has never been quantified.

A potentially important aspect of sea urchin-mediated bioerosion is food availability in both subtidal and intertidal habitats. Through their feeding habits, sea urchins control the balance between the alternative stable states of kelp forests and urchin barrens: two habitats exhibiting vastly different levels of primary productivity/food availability (Scheibling 1986; Harrold and Pearse 1987; Watanabe and Harrold 1991; Bonaviri et al. 2012; Flukes et al. 2012; Suskiewicz and Johnson 2017). Higher food availability is associated with lower sea urchin populations, as decreases in local sea urchin abundance (as in kelp forests) controls the presence of macroalgae (Simenstad et al. 1978; Palacin et al. 1998; Bronstein and Loya 2014; Pert et al. 2018). In intertidal habitats, kelp and other algae that wash ashore can provide a significant food source for sea urchin populations and other invertebrates (Rodríguez 2003; Notman et al. 2016), with algae washing ashore both close to its original source (López et al. 2017; Schreiber et al. 2020) or longer distances (Hinojosa et al. 2011). However, the timing and amount of drift algae may depend on several factors such as season, currents, and winds (Hinojosa et al. 2011; Hawes et al. 2017; López et al. 2017; Schreiber et al. 2020). Drift algae is also important for sea urchins in barren areas (Harrold and Reed 1985). Despite the omnipresence of *S. purpuratus* in nearshore habitats and its voracious appetite for macroalgae, the relationship between bioerosion and food availability remains unknown. It is reasonable to assume that when sea urchins are grinding and processing algae, the teeth are not abrading the rocks they are attached to and thus do not simultaneously both bioerode substratum and masticate algae.

A second unexplored aspect of sea urchin bioerosion is the relative rates of tooth vs spine abrasion. Bioerosion rates of sea urchins vary with the lithology of the substrate, and along the California coast, the purple sea urchin is found on substrates that erode at different rates (sandstone > mudstone > granite; Russell et al 2018). Differences in sea urchin-mediated bioerosion rates among substrates correspond to different levels of sediment output in these rocky habitats. Across their geographic range, purple sea urchins can produce as much sediment as the discharge (sediment load) of many rivers (in excess of 10^6^ t yr^−1^; Russell et al. 2018). However, no studies have assessed the role of spinal abrasion on these different substrates. The magnitude of spinal abrasion may prove to be significant and likely varies with substrate type. If sea urchins abrade substrate with their spines, then several more factors could be significant in bioerosion studies (i.e., urchin size, movement, spine characteristics). Moreover, demonstrating and quantifying the bioerosion of rocky substrates by sea urchin spines are essential to understanding the mechanisms of bioerosion and ecology of intertidal and rocky reefs.

In this study, we tested the hypothesis that sea urchins with less available food would exhibit increased bioerosion rates across substrates compared to sea urchins with higher food availability. We also hypothesized that sandstone would exhibit higher bioerosion rates than mudstone based on previous work (Russell et al. 2018), and that spinal abrasion would account for at least a portion of the material eroded. Our experiment addresses the question of how food availability affects bioerosion rates across rocky habitats with different substrates. The results of this experiment provide insight on the mechanisms of bioerosion as well as how changes in algal availability may affect bioerosion and sediment production in other species and habitats.

## Methods

### Field collection

We collected *S. purpuratus* from Bean Hollow Beach, California (37°13′N 122°24′W) and Palomarin Beach in Bolinas, California (37°55′N 122°44′W), USA, in June 2018 and immediately shipped them to the laboratory at Villanova University. At each site, we collected 10 sea urchins in approximately the same range of sizes (four ≤ 40 mm and six > 40 mm test). Individuals were then acclimated in the recirculating seawater system for 1 week before treatments began. Sea urchins collected from Bean Hollow Beach were kept on their native sandstone substrate, and individuals from Palomarin on their native mudstone, for the entirety of the 11-week experiment.

### Experimental set-up

Sea urchins were kept in a 1200-L recirculating seawater system with relatively constant temperature (range 11.2–12.3°C) and salinity (range 30.6–32.3 ppt). We monitored seawater temperature, salinity, and pH daily, and measured water quality (ammonia, magnesium, calcium, nitrate, nitrite, phosphate, and alkalinity levels) at least once a week. We kept water quality measurements similar to suggested ranges (Basuyaux and Mathieu 1999; Holmes-Farley 2004).

We drained and cleaned the sea table at least every other day to remove feces and any excess kelp and detritus.

The effect of substrate lithology on bioerosion rates was assessed by placing sea urchins on natural substrates (hereafter rock units) that were collected from the same sites as the sea urchins. We used a wet masonry saw equipped with a 35.6-cm diamond blade to cut larger sandstone blocks from Bean Hollow Beach into square pieces. The diamond blade saw shattered the mudstone so we used a small, flat, cobble mudstone for each rock unit. We slightly disturbed rock surfaces with a metal grater to standardize the rock unit surfaces. We constructed 11.5 × 11.5 cm^2^ units and embedded the sandstone and mudstone rock into a mixture of marine epoxy and pea gravel leaving the flat rock surface exposed. Units were air-dried for at least 24 h before small PVC segments were glued to the bottom of the units to fit in a large PVC (Polyvinyl chloride) grid in the sea table. After marine epoxy and glue had dried, rock units were rinsed and soaked in deionized water for 24 h. Rock units were then placed in drying ovens until weights of the units were stable. We secured plastic mesh cages to each unit extending above the water level to prevent escape and restrain sea urchins to the rock surface (Supplementary Fig. S1).

We installed a PVC grid system to keep units in place and an overhead PVC sprinkler system powered by pumps to generate water flow to each unit. We rotated the position of units on the grid every week to account for variation in flow rates of the sprinkler system. The average flow rate for one sprinkler delivering water to one unit was 1.29 ± 0.05 L/min.

### Effects of substrate and food availability on bioerosion rates

We used a 2 × 2 factorial design with the factors substrate (sandstone and mudstone) and feeding (fed and starved). There were five replicates in each of the four treatments (substrate and feeding combination)—with two sea urchins from the ≤40 mm and three from the >40 mm test size class. The largest individual in the Mudstone:Fed treatment died prior to the experiment (*n* = 4 for that treatment). There were three controls for each substrate, a rock unit with an attached cage but no sea urchin, allowing us to account for any unit weight change not due to sea urchin presence/bioerosion.

In the “fed” treatment, sea urchins always had food present. They were supplied with commercially available kelp (Laminaria, Wel-Pac brand) *ad-libitum* (typically a few grams of kelp—depending on urchin size and consumption rates in previous feeding periods—every 2 days). In the “starved” treatment, sea urchins were fed just once per week (mass of kelp determined in same manner as “fed” sea urchins) during a 24 h period—and then kelp was removed. Although purple sea urchins can survive extended periods without food (e.g., sea urchin barrens), the feeding treatments nevertheless represent very different levels of food availability (a seven-fold difference). The dehydrated kelp was rehydrated in deionized water for 5 days and used as food.

Final weight of rock units was determined by soaking them in deionized water overnight and drying them until the weight was constant. Net bioerosion was calculated as the difference in rock unit weight before and after the experiment and standardized bioerosion was calculated by dividing the net bioerosion by sea urchin diameter. Annual bioerosion rates were calculated using the net bioerosion rates.

We created the “net change” metric to quantify the amount of bioerosion per unit while taking into account the change in mass of the control units without sea urchins, i.e., change not due to bioerosion (Equation 1).


(1) \begin{eqnarray*}
{\mathrm{Net \,\,\mathit{\mathrm{change}} \,\,}} = {\mathrm{ \,\,}}{{D}_{if}} + \left( {{{W}_i}{\mathrm{ \,\,}} \bullet {\mathrm{ \,\,}}{{C}_{if}}/{{C}_i}} \right)
\end{eqnarray*}

${{D}_{if}}$ represents the difference between initial and final weight of a rock unit with a sea urchin (change after 11 weeks). ${{W}_i}$ represents the initial weight of the unit. ${{C}_{if}}$ is the difference in initial and final weights of the control units for a given substrate, and ${{\mathrm{C}}_i}$ then is the initial weight of the control units. ${{C}_{if}}/{{C}_i}$ represents the % change in the control units for a particular substrate without sea urchins (non-bioerosion change). Since there were three controls for each substrate, we used the mean ${{C}_{if}}/{{C}_i}$ of the three controls for each substrate.

We tested for differences between substrates or feeding treatments using net change and net change divided by sea urchin diameter. We divided net change by sea urchin diameter to assess if sea urchin size affected bioerosion rates, which is particularly important if spinal abrasion is a significant factor in bioerosion.

### Distinguishing spine from tooth bioerosion

Quantifying the amount of bioerosion from teeth vs spines requires distinguishing the respective sources of the sediment eroded from the substratum. We accomplished this by isolating individuals in enclosed containers (buckets), for two different 24-h periods (separated by 1 week), on different substrates (rock vs glass), and collecting all the material in the container at the end of the period. The buckets were thoroughly cleaned before the start of the isolation and no food was provided. We assumed that the rock material that passes through the digestive tract is material eroded by the teeth and present in the feces (Russell et al. 2018). The other rock material eroded is from abrasion by the spines. One 24-h isolation was their natural rock substrate where the sediment collected was from both teeth (fecal production) and spine abrasion. The other isolation was on a flat glass substrate where there was no sediment produced by spine abrasion, but the sea urchins produced feces that contained sediment eroded (and ingested) from their rock substrates prior to the isolation. Under both conditions, the sea urchins continued to produce fecal material that contained sediment scraped by their teeth (and ingested—passage of material through the digestive system is approximately 2 days; Lawrence and Klinger 2001). The difference between these two settings yielded the amount of sediment from spine abrasion: on rock (teeth + spine abrasion)—on glass (teeth abrasion only).

To distinguish fecal material (organic) from rock (mostly inorganic—see below), we measured the dry and ash weights of all the material collected for each sea urchin in both settings. After we removed the sea urchin and substrate from the bucket, we allowed the feces and rock material to settle. This “waste” was rinsed three times with deionized water in the bucket. On the third and final rinse, we decanted off the salt-less water and placed the waste material into a preweighed crucible. After the waste settled in the crucible and excess water was removed, crucibles were placed in a drying oven (∼55°C) for a minimum of 48 h and then dry-weighed. We then ashed samples at 500°C for 4 h to measure inorganic content. We calculated the inorganic content of the waste material as a percentage:


(2) \begin{eqnarray*}
\frac{{{\mathrm{Ash \,\,\mathit{Weight}}}}}{{{\mathrm{Dry \,\,\mathit{Weight}}}}}{\mathrm{ \,\,}} = {\mathrm{ \,\,\% \,\,\mathrm{inorganic} \,\,\mathrm{content}}}
\end{eqnarray*}

We assumed that the contribution of inorganic material from kelp fecal material alone (sans rock) was the same in both settings and across rock types.

Both sedimentary rock types have a small, but different, organic component, and thus a different % inorganic content. To account for this (organic) rock material that was burned off and not present as ash weight, we measured the % inorganic content of both types of rocks. Rock samples were ground with a mortar and pestle, and both ash and dry weights measured.

We defined Sp as the fraction of the inorganic content of samples from spine abrasion, so [1 − Sp] is the fraction from tooth abrasion. For each rock type, we used the % inorganic of the rock (I_ROCK_), the mean of the inorganic % on glass (M%_ON-GLASS_ = from teeth abrasion only), and the Mean of the inorganic % on rock (M%_ON-ROCK_ = from teeth and spine abrasion), to solve for Sp and 1 − Sp for sandstone and mudstone substrates:


(3) \begin{eqnarray*}
{\mathrm{M}}{\% _{{\mathrm{ON - ROCK}}}} &=& \left( {{\mathrm{Sp}} \bullet {{\mathrm{I}}_{{\mathrm{ROCK}}}}} \right)\\
&&+ \left( {\left[ {1 - {\mathrm{Sp}}} \right] \bullet {\mathrm{M}}{\% _{{\mathrm{ON - GLASS}}}}} \right)
\end{eqnarray*}

### Effects of food availability and substrate on feeding rates and growth

For all replicates, and each feeding period, during the 11 weeks, we quantified the amount of kelp consumed. Kelp was cut, pressed between two paper towels to dry and then weighed. Over the course of the experiment (for each sea urchin), we calculated consumption rates (grams of kelp consumed per 24 h) by comparing the weight of kelp before and after each feeding regime.

We used two measures to assess the effects of feeding and substrate on growth. Before and after the 11 weeks, we measured test diameter and weighed the sea urchins and analyzed the difference metric in both variables.

### Statistical analysis

We conducted statistical analyses and generated graphs in R (version 3.4.3, www.r-project.org). We used factorial analyses to analyze the 2 net change metrics (net bioerosion and net bioerosion/diameter), difference in % inorganic content trials of waste collection, % diameter change, and weight (Table 1). We tested for normal distribution of residuals with Shapiro’s test and homogeneity of variance was confirmed with a Bartlett’s homoscedasticity test. Data were transformed with log10 or natural log (ln) when normality or homogeneity assumptions were not met (Supplementary Table S2). We used an analysis of covariance (ANCOVA) to analyze consumption rates with respect to sea urchin wet weight and feeding treatments.

**Table 1.** The results of all factorial ANOVA tests conducted in this study

	Treatment factors		
Response variable	Substrate	Feeding	Interaction
Bioerosion	*F*_1,15_ = 0.912 *P* = 0.355	*F*_1,15_ = 0.343 *P* = 0.567	*F*_1,15_ = 0.281 *P* = 0.604
$\frac{{{\mathrm{Bioerosion}}}}{{{\mathrm{Diameter}}}}$	*F*_1,15_ = 1.467 *P* = 0.245	*F*_1,15_ = 0.547 *P* = 0.471	*F*_1,15_ = 0.702 *P* = 0.415
Inorganic % (Rock—Glass)	*F*_1,15_ = 5.045 *P* = 0.0402	*F*_1,15_ = 0.006 *P* = 0.939	*F*_1,15_ = 0.704 *P* = 0.415
Growth			
Weight	*F*_1,14_ = 12.710 *P* = 0.00311	*F*_1,14_ = 8.357 *P* = 0.0119	*F*_1,14_ = 1.873 *P* = 0.193
Diameter	*F*_1,15_ = 2.060 *P* = 0.1717	*F*_1,15_ = 6.897 *P* = 0.0191	*F*_1,15_ = 0.384 *P* = 0.5446

## Results

At the start of the experiment, there were no differences in either sea urchin test diameters (substrate: *F*_1,15_ = 0.00, *P* = 0.987; feeding: *F*_1,15_ = 0.085, *P* = 0.775; interaction: *F*_1,15_ = 0.030, *P* = 0.864) or wet weights (substrate: *F*_1,15_ = 0.008, *P* = 0.929; feeding: *F*_1,15_ = 0.063, *P* = 0.805; interaction: *F*_1,15_ = 0.048, *P* = 0.829) among the four experimental groups. Over the 11 weeks, there were slight losses in dry weights of the control rock units without sea urchins: ${{\mathrm{C}}_{if}}/{{\mathrm{C}}_i}$ was 0.2880% ± 0.0233% for sandstone and 0.4702% ± 0.0907% for mudstone (mean ± SE).

### Bioerosion

**Fig. 1:**
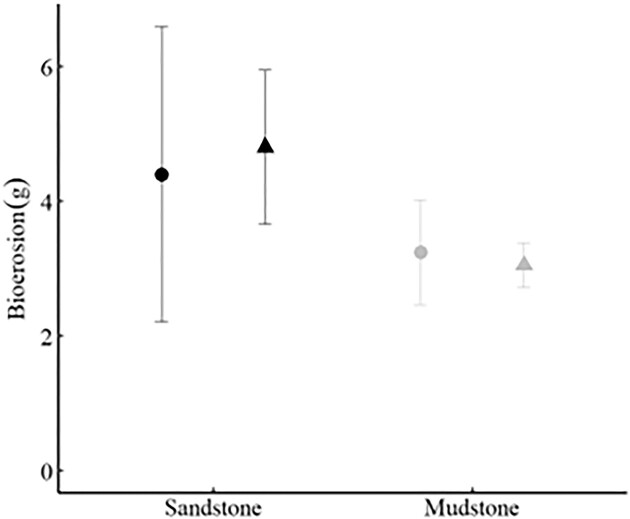
Bioerosion (mean ± SE) over 11 weeks under two feeding treatments (fed-circles; starved-triangles) and on two different substrates (sandstone-black; mudstone gray). There was no significant effect of either feeding or substrate (Feeding: *F*_1,15_ = 0.343, *P* = 0.567; Substrate: *F*_1,15_ = 0.912, *P* = 0.355).

There were no differences in either net, or standardized, bioerosion rates between substrates or feeding treatments, and no interaction between treatments (Fig. 1 and Table 1). The feeding treatment had no effect on bioerosion as the mean bioerosion rates were nearly identical between fed and starved sea urchins (combining rock types—fed: 3.89 ± 1.22 g; starved: 3.93 ± 0.63 g [mean ± SE]). Although there was no significant difference between rock substrates, sandstone rock units had mean bioerosion rates 46% higher than mudstone rates (combining feeding treatments—sandstone: 4.60 ± 1.17 g; mudstone: 3.14 ± 0.37 g [mean ± SE]; Table 2). This difference is consistent with previous bioerosion studies that were conducted over 1 year rather than 11-weeks (Russell et al. 2018).

**Table 2.** Bioerosion rates (mean ± SD) measured in this study (11 weeks) and predicted bioerosion rates over 1 year

Bioerosion	Sandstone	Mudstone
Experimental total (g × 11wk^−1^)	4.60 ± 3.69	3.14 ± 1.10
Spines (g × 11wk^−1^)	1.43	0.04
Teeth (g × 11wk^−1^)	3.17	3.10
Estimated total (g × yr^−1^)	21.76	14.84
Spines (g × yr^−1^)	6.77	0.19
Teeth (g × yr^−1^)	14.99	14.65

The first row of data represents predicted total bioerosion of substrate (g × yr^−1^), and the second and third rows are the fraction of that total bioerosion due to spines and teeth, respectively. Feeding treatment was not included since there was no difference in bioerosion rates.

### Tooth and spine bioerosion

**Fig. 2:**
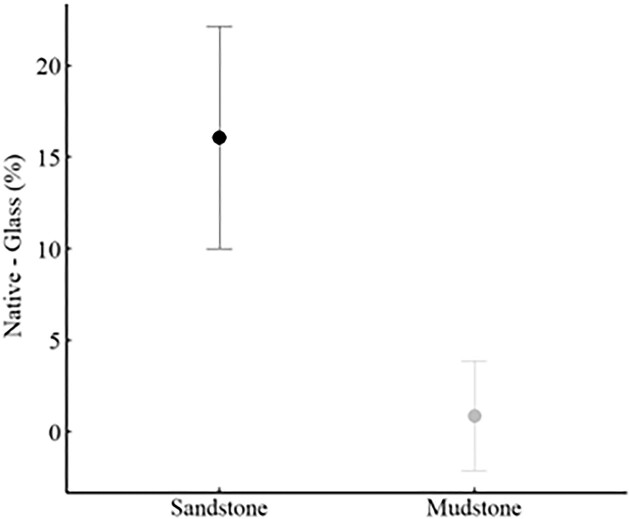
Mean (±SE) difference in inorganic content (Rock-Glass) % of 24 h waste collection trials. The same sea urchins were placed for 24 h on their native (rock) and a glass substrate and the difference in % inorganic content was used to estimate the spinal abrasion. Results grouped by native substrate. There was a significant difference in means (*t*_1,17_ = 2.27, *P* = 0.041).

Sandstone and mudstone rock samples had inorganic contents of 98.88% ± 0.14% and 95.31% ± 0.72%, respectively ([mean ± SE]; *n* = 15 for each rock type). The waste collected from the 24-h isolation treatments exhibited a higher % inorganic content on rock substrates (sandstone: 63.28% ± 7.56%, mudstone 30.84% ± 8.22% [mean ± SE]) than on glass trials (sea urchins from sandstone: 47.22% ± 5.13%, sea urchins from mudstone 29.99.% ± 5.60% [mean ± SE]). Using these values, and solving equation 3 (above) yields Sp (fraction of erosion from spine abrasion) values of 31.09% for sandstone and 1.30% for mudstone. This difference in source of bioerosion is confirmed by the significant effect of substrates in the difference (rock—glass, for each sea urchin) in % inorganic (*F*_1,15_ = 5.045, *P* = 0.040; Fig. 2) but not feeding treatments (*F*_1,15_ = 0.006, *P* = 0.94; Table 1).

In sandstone, 31.09% of total bioerosion was due to spinal abrasion, in stark contrast to mudstone where it was only 1.30%. To annualize the mean rock unit weight loss, bioerosion can be multiplied by a factor of 4.73 (the rate of 11 weeks to equal 52 weeks). These annual rates make clear the differences in substrate bioerosion rates and the sources: over the course of 1 year, bioerosion by teeth is nearly identical between substrates (14.99 g for sandstone and 14.65 g for mudstone; Table 2). However, the nearly 50% difference in total bioerosion (21.76 g for sandstone and 14.84 g for mudstone) is due to spines (6.77 g for sandstone and 0.19 g for mudstone; Table 2).

### Rates of food consumption and growth

**Fig. 3:**
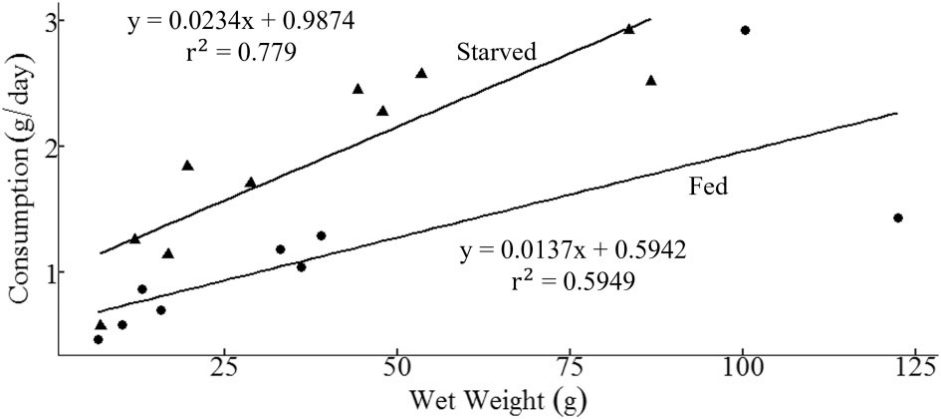
Standardized sea urchin consumption rates (g of kelp) over 24 h under two different treatments: Fed constantly (circles) or starved 6 days per week (triangles). Points are means for each sea urchin (wet weight of sea urchin at start) over the 11 week experiment. Within feeding groups, substrates (sandstone and mudstone) were combined.

Based on kelp consumption over the 11 weeks, we standardized (to g/24-h feeding period) for each sea urchin. Although an ANCOVA revealed no significant difference in the slopes of consumption rates using wet weight as the covariate (*F*_1,15_ = 0.2772, *P* = 0.6063); there was a significant difference between the two groups with starved sea urchins having higher consumption rates than fed over the course of the 11 weeks (Fig. 3, *F*_1,16_ = 15.58, *P* = 0.0012).

**Fig. 4:**
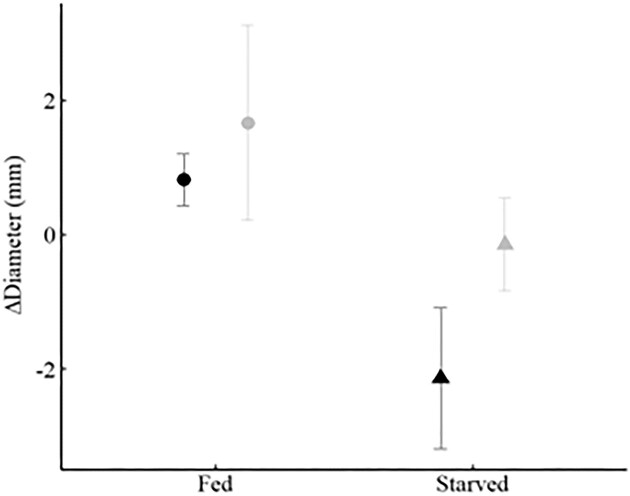
Growth rate measured as change in diameter (mean ± SE) over 11 weeks under two feeding treatments (fed-circles; starved-triangles) and on two different substrates (sandstone-black; mudstone-gray). Feeding treatment was significantly different (*F*_1,15_ = 6.897, *P* = 0.0191) but not substrate (*F*_1,15_ = 2.060, *P* = 0.1717).

**Fig. 5:**
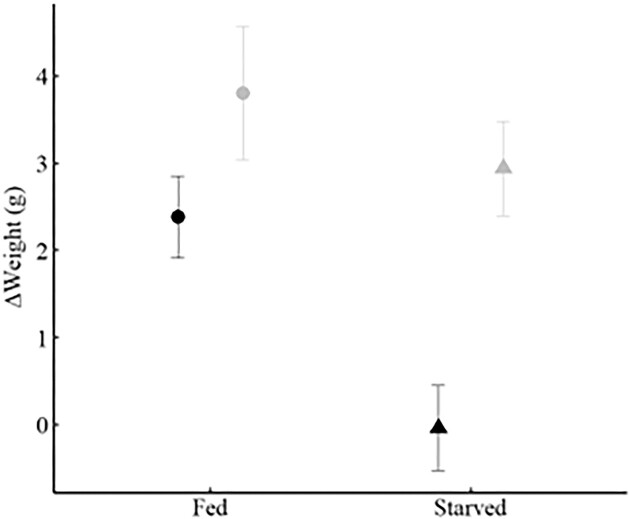
Growth rate measured as change in weight (mean ± SE) over 11 weeks under two feeding treatments (fed-circles; starved-triangles) and on two different substrates (sandstone-black; mudstone-gray). Both feeding treatment (*F*_1,14_ = 8.357, *P* = 0.0119) and substrate (*F*_1,14_ = 12.710, *P* = 0.003) were significantly different.

In growth rates, the factorial ANOVA showed there was no interaction in either change in diameter (*F*_1,15_ = 0.384, *P* = 0.5446) or in change in weight (*F*_1,14_ = 1.837, *P* = 0.193; note, we failed to weigh one sea urchin at the end of the experiment in the Sandstone:Starved treatment so the df’s for this *F* value are 1,14 instead of 1,15). Both change in diameter and change in weight showed that fed sea urchins grew significantly more than starved sea urchins (Table 1, Figs. 4 and 5). The trend in both measures of growth showed that sea urchins on mudstone grew faster than sea urchins on sandstone, but the pattern was only statistically significant for change in weight (*F*_1,14_ = 12.710, *P* = 0.003) and not statistically significant for change in diameter (*F*_1,15_ = 2.060, *P* = 0.1717).

## Discussion

Bioerosion rates of fed vs starved *Strongylocentrotus purpuratus* were nearly identical (Fig. 1, Table 1) and we failed to reject the null hypothesis of no difference in bioerosion between feeding treatments. This result strongly suggests that sea urchins occurring in different habitats (kelp forests or sea urchin barrens; intertidal areas with high vs low drift algae; sea urchins inhabiting pits with different food availability) will have similar bioerosion rates. Because most of bioerosion is from the action of the teeth on the substrate, we infer that the mechanics of the teeth abrading the rock are not different in the presence vs the absence of algae. As sea urchins feed, they simultaneously bioerode the rocky surface and masticate algae.

There was also no significant difference between rock types in either net bioerosion or bioerosion adjusted for size of the sea urchins (Fig. 1, Table 1). This result is in contrast to what Russell et al. (2018) found using rocks (and sea urchins) from the same sites. They found that bioerosion was significantly higher on sandstone. An important difference between our work and the Russell et al. (2018) study is time; their study lasted one year as compared to 11 weeks in our study. However, the annualized rates (Table 2) suggest that bioerosion differentially impacts the two different rock types.

Our study quantified, for the first time, the proportion of bioerosion from teeth and spines, and that the tooth:spine ratio was very different between the two types of rocks. Furthermore, teasing apart the amount of bioerosion from the two sources showed that the rates of bioerosion from teeth were nearly the same between the two types of substrates. We can only speculate about why the teeth seem to have the same impact on the two rocks, whereas the spines seem to erode more sandstone than mudstone. The tips of the teeth are sharp (and self-sharpening), whereas the tips of the spines are more blunt. The very sharp point of the tips of the teeth have been described as “a remarkable grinding tool” (Ma et al. 2009) and the teeth probably grind and bore both sandstone and mudstone with an equal effect. The sedimentary particles comprising mudstone are much smaller than the larger granular sandstone, giving mudstone a smoother surface. It is possible that as the spines rub against the surface of mudstone, they slide smoothly and do not abrade much material. In contrast, the larger, granular sedimentary particles of the sandstone, may become dislodge and abrade away from the matrix as the tips of the spine sweep across the surface. Regardless of the mechanics, it is clear that the teeth and spines differentially impact the surfaces of substrates.

Spinal abrasion accounted for more than 30% of the total bioerosion on the sandstone substrate, while it had almost no effect on mudstone. Spinal abrasion rates may increase with increased sea urchin movement that could be influenced by a variety of factors such as variability in predators, food, and aggregation (Russo 1979; Rodriguez and Ojeda 1998; Parnell et al. 2017; Spyksma et al. 2017). Additionally, sea urchins in barren grounds tend to occur in higher densities while also exhibiting longer spines (Ling and Johnson 2009). Another aspect that should be considered in estimating the role of spinal abrasion is the shape of the erodible surfaces (two-dimensional vs three-dimensional). Our experimental units were flat, restricting the influence of spine bioerosion to spines located toward the oral side of the sea urchin. In pits, however, sea urchins could potentially use spines located on the ambitus and aboral sides, increasing the abrasion.

Although we predicted that sea urchins with unlimited access to food would grow faster than sea urchins restricted to feeding one day a week, we were not sure this result would be evident in just 11 weeks. Even over this relatively short period, fed sea urchins exhibited greater changes in both diameter and weight. However, we did not expect that substrate would have an effect on growth rate. The pattern of increased growth rate on mudstone was the same for both diameter and weight; however, it was more evident (and statistically significant) in terms of weight. Sea urchins on mudstone gained 3.37 ± 0.45 g (mean ± SE) compared to 1.30 ± 0.53 g (mean ± SE) on sandstone. What we found more surprising is that starved sea urchins on mudstone (2.94 ± 0.54 g [mean ± SE]) gained more weight than fed sea urchins on sandstone (2.38 ± 0.47 g [mean ± SE]).

There are at least two explanations of this unusual result—or at least contributing factors—the organic content of mudstone and possible differences in the biofilms associated with the different rocks. The ash weights of the rocks showed that mudstone has more than 4× the organic content of sandstone (4.69% compared to 1.12%). This difference combined with potential differences in biofilms may account for our results and strongly suggests that growth rates in the field may not be the sole product of food availability. Further work on this tantalizing result is warranted.

In our experiment, we only studied two factors (food availability and substrate) in bioerosion, but kelp forests, sea urchin barrens, and other rocky temperate reefs where sea urchins are key constituents exhibit additional ecological differences. Factors such as sea urchin density, spine length, movement, and even amount of commercial harvesting (Harrold and Reed 1985; Ling and Johnson 2009; Pert et al. 2018) likely affect bioerosion. Population density influences sea urchin behavior, as sea urchin movement decreases with increased population density but grazing rates increase with density (Dumont et al. 2007; Lauzon-Guay and Scheibling 2007). These factors, combined with differences in lithology, likely result in more variation in bioerosion than documented in this study.

## Supplementary Material

icae060_Supplemental_File

## Data Availability

The DOI for the data files associated with this publication is: https://doi.org/10.6084/m9.figshare.25929268.v1.
