# Supplementary material for: Effects of Rock Type and Food Availability on Bioerosion by the Purple Sea Urchin, Strongylocentrotus purpuratus
Source: Integr Comp Biol. 2024 Jun 3;64(6):1527–35. doi: 10.1093/icb/icae060 (PMC11659677; doi:10.1093/icb/icae060)
Supplement: icae060_Supplemental_File [file icae060_supplemental_file.pdf]

Supplementary Material

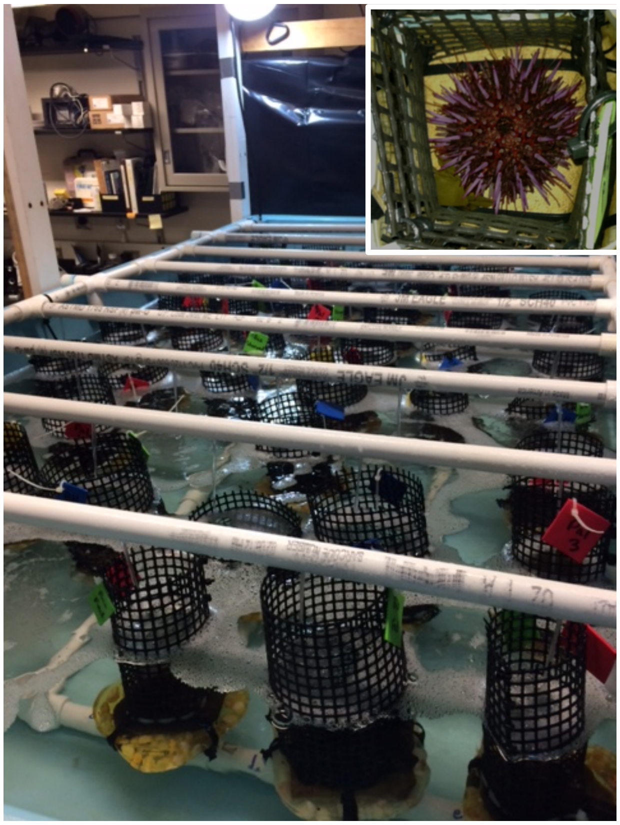

Fig. S1. Sea table set-up. The overhead PVC sprinkler system, PVC grid base, and individual units with their accompanying cages are shown (n = 25; units from another experiment are also present). The inset photograph in the upper right corner is an overhead view of one of the units with a sea urchin on a sandstone substrate

Supplementary Table 1. Assumption results of each final analysis.

Analyzing	Normality	Homoscedasticity	Transformation
Consumption Rates	Wet Weight:<br>W = 0.95973<br>p = 0.5671<br>Consumption:<br>W = 0.93958<br>p = 0.2591	K^2 = 3.1022<br>p = 0.07818	Natural log (ln)
Net Change	W = 0.90712<br>p = 0.06556	Substrate:<br>K^2 = 3.4462<br>p = 0.0634<br>Feeding:<br>K^2 = 2.0909<br>p = 0.1482	Log10
$\frac{\text{Net Change}}{\text{Diameter}}$	W = 0.97357<br>p = 0.8448	Substrate:<br>K^2 = 1.5988<br>p = 0.2061<br>Feeding:<br>K^2 = 0.10542<br>p = 0.7454	Log10
Inorganic %<br>(Rock – Glass)	W = 0.92481<br>p = 0.1389	K^2 = 2.3758<br>p = 0.1232	Log10 + 10
**Growth**			
Diameter	W = 0.97384<br>p = 0.8497	Substrate:<br>K^2 = 0.44585<br>p = 0.5043<br>Feeding:<br>K^2 = 0.18737<br>p = 0.6651	None
Weight	W = 0.9315<br>p = 0.2063	Substrate:<br>K^2 = 1.4866<br>p = 0.2227<br>Feeding:<br>K^2 = 1.1315<br>p = 0.2875	None
